# Data on coffee composition and mass spectrometry analysis of mixtures of coffee related carbohydrates, phenolic compounds and peptides

**DOI:** 10.1016/j.dib.2017.05.027

**Published:** 2017-05-17

**Authors:** Ana S.P. Moreira, Fernando M. Nunes, Cristiana Simões, Elisabete Maciel, Pedro Domingues, M. Rosário M. Domingues, Manuel A. Coimbra

**Affiliations:** a QOPNA, Department of Chemistry, University of Aveiro, 3810-193 Aveiro, Portugal; b CQ-VR, Chemistry Research Centre, Department of Chemistry, University of Trás-os-Montes e Alto Douro, 5001-801 Vila Real, Portugal; c CESAM, Department of Biology, University of Aveiro, 3810-193 Aveiro, Portugal

**Keywords:** Coffee, Carbohydrates, Polysaccharides, Phenolics, Mass spectrometry, Melanoidins

## Abstract

The data presented here are related to the research paper entitled “Transglycosylation reactions, a main mechanism of phenolics incorporation in coffee melanoidins: inhibition by Maillard reaction” (Moreira et al., 2017) [1]. Methanolysis was applied in coffee fractions to quantify glycosidically-linked phenolics in melanoidins. Moreover, model mixtures mimicking coffee beans composition were roasted and analyzed using mass spectrometry-based approaches to disclose the regulatory role of proteins in transglycosylation reactions extension. This article reports the detailed chemical composition of coffee beans and derived fractions. In addition, it provides gas chromatography–mass spectrometry (GC–MS) chromatograms and respective GC–MS spectra of silylated methanolysis products obtained from phenolic compounds standards, as well as the detailed identification of all compounds observed by electrospray mass spectrometry (ESI-MS) analysis of roasted model mixtures, paving the way for the identification of the same type of compounds in other samples.

**Specifications Table**

**Table:** 

Subject area	*Chemistry*
More specific subject area	*Composition of coffee and mass spectrometry analyses of coffee related carbohydrates, phenolic compounds and peptides*
Type of data	*Tables and figures*
How data was acquired	*Methanolysis products were analyzed by GC–MS (Trace-GC with Polaris Q MS, Thermo-Finnnigan, San Jose, CA)*
	*Content of chlorogenic acids, caffeine, adsorbed phenolics, and phenolics released by alkaline saponification and alkaline fusion was obtained by HPLC (Dionex Ultimate 3000*, *Thermo, Waltham, MA);*
	*Content of sucrose, glucose and fructose was determined by anion exchange chromatography (ICS 3000, Dionex);*
	*Total sugars were determined by GC-FID (Trace GC, Thermo-Finnigan);*
	*Protein content was determined using a carbon-nitrogen/protein analyzer (PRIMACS, Skalar Analytical B.V., Breda, The Netherlands);*
	*Roasted coffee powder colors were measured with a CR-300 Minolta chroma meter (Tokyo, Japan);*
	*HPLC-ESI-MS analysis used a Waters Alliance 2690 HPLC system (Milford, MA) coupled to the LXQ linear ion trap (LIT) mass spectrometer (Thermo Fisher Scientific Inc., Waltham, MA);*
	*Direct ESI-MS analyses were also performed using LIT mass spectrometer;*
	*High resolution and high mass accuracy measurements were performed on a LTQ-Orbitrap XL mass spectrometer (ThermoFisher Scientific, Germany).*
Data format	*Analyzed*
Experimental factors	*Roasted model mixtures and coffee beans*
Experimental features	*Chemical characterization and identification of the changes induced by roasting*
Data source location	*Robusta coffee beans from India*
	*Arabica coffee beans from Honduras*
	*Commercial standards of carbohydrates, phenolic compounds and peptides*
Data accessibility	*Data is provided with this article*

**Value of the data**

• Detailed chemical characterization of Arabica and Robusta coffee beans and derived fractions is able to be compared with data from other authors when profiling the phenolic compounds incorporated in melanoidins.
• GC–MS data of methanolysis products of phenolic compounds standards provide information on the efficiency and linkages cleaved by methanolysis and are the basis for their identification in real samples.
• Mass spectrometry data on the roasting-induced compounds formed from model mixtures mimicking coffee bean composition are valuable for the identification of the same type of compounds in roasted coffee, but also other complex roasted carbohydrate-rich matrices.

## 1 Data

**Fig. 1:**
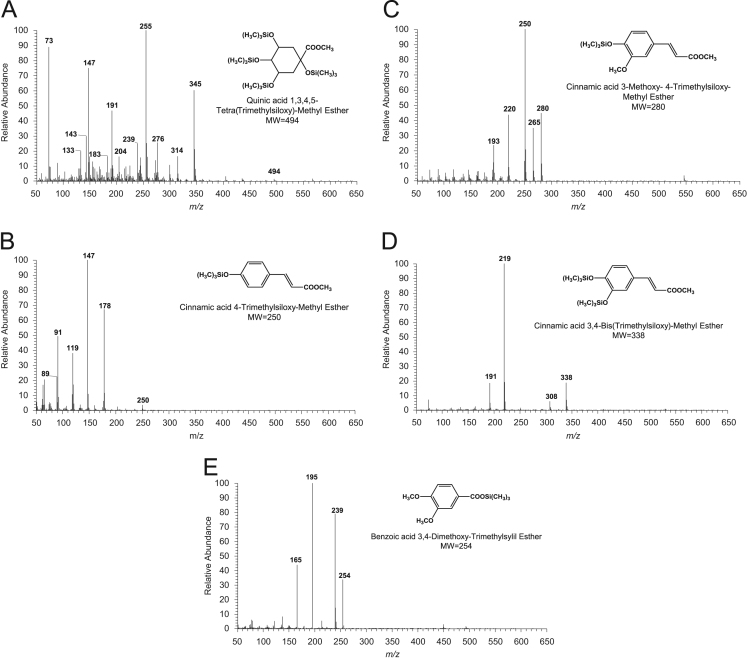
Structures and GC–MS spectra obtained from standards after silylation of the respective products released by methanolysis: A) quinic acid derivative; B) *p*-coumaric acid (4-hydroxycinnamic acid) derivative; and C) ferulic acid derivative. D) caffeic acid derivative; and E) veratric acid derivative.

**Fig. 2:**
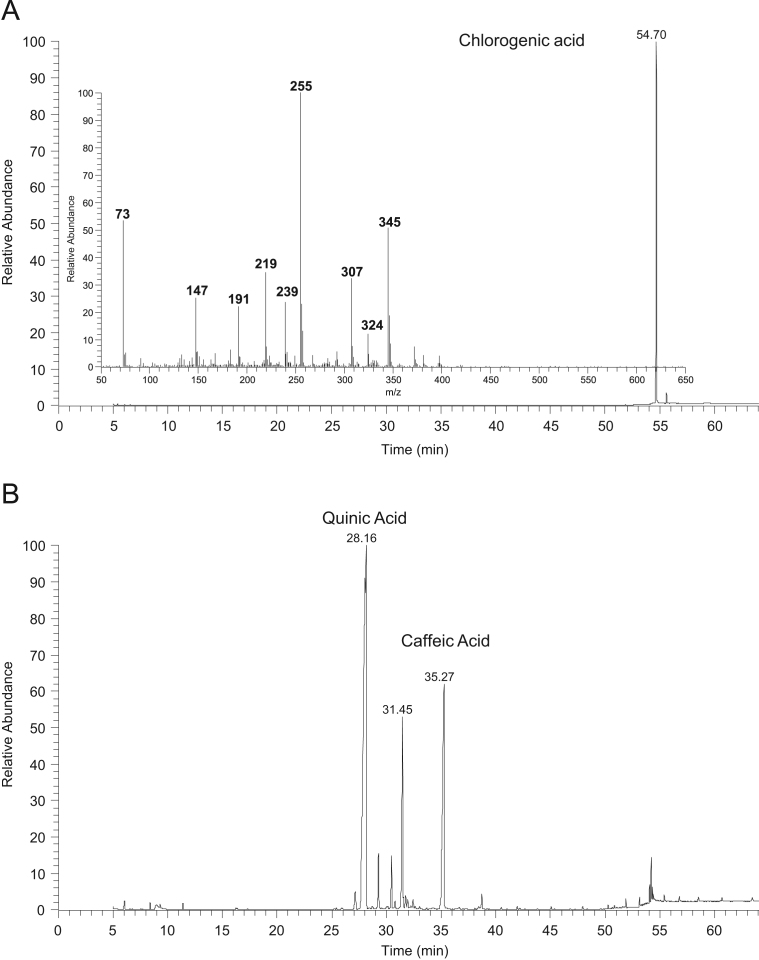
GC–MS chromatograms of A) 5-*O*-caffeoylquinic acid after silylation with the respective mass spectrum of the peak at the retention time 54.70 min, and B) products of 5-*O*-caffeoylquinic acid methanolysis after silylation.

**Fig. 3:**
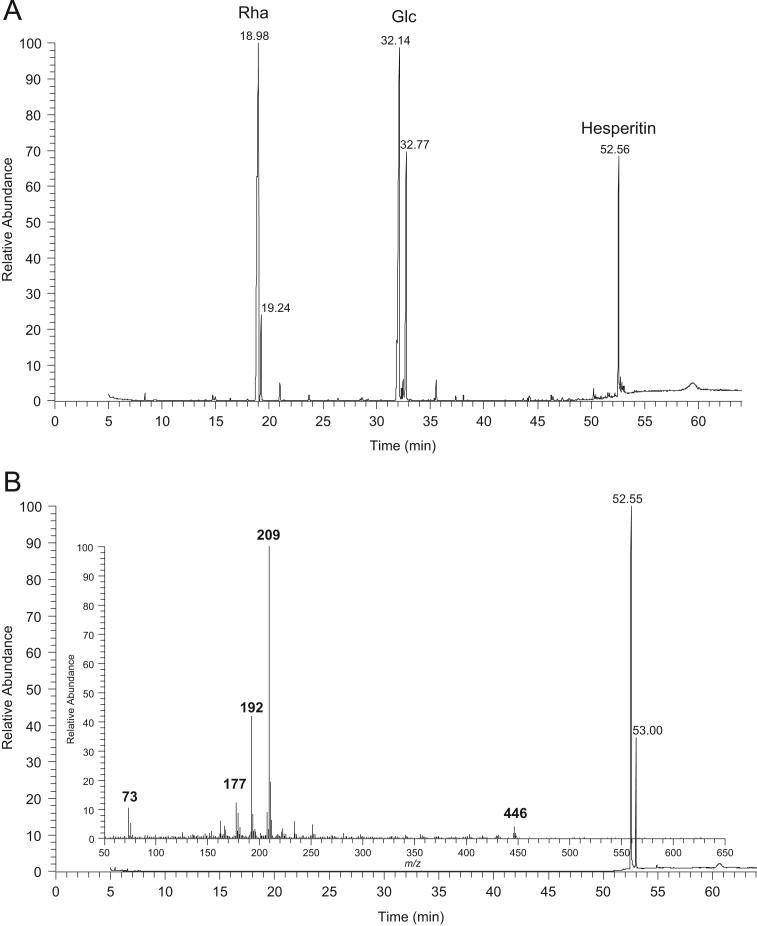
GC–MS chromatograms of A) products of hesperidin methanolysis after silylation and B) hesperetin after silylation with the respective mass spectrum of the peak at the retention time 52.55 min.

**Fig. 4:**
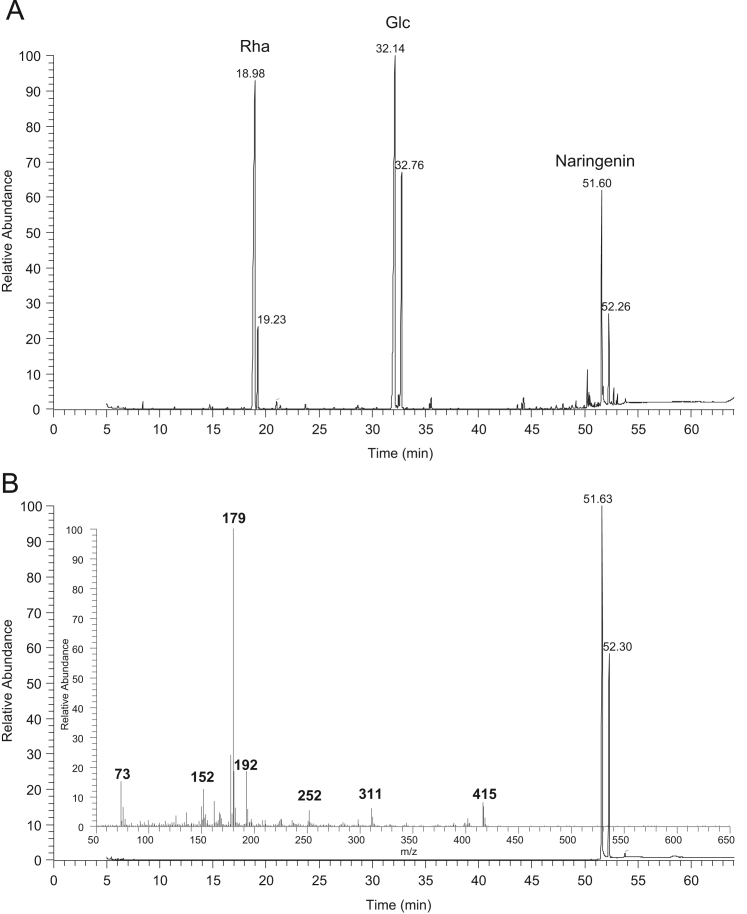
GC–MS chromatograms of A) products of naringin methanolysis after silylation and B) naringenin after silylation with the respective mass spectrum of the peak at the retention time 54.63 min.

**Fig. 5:**
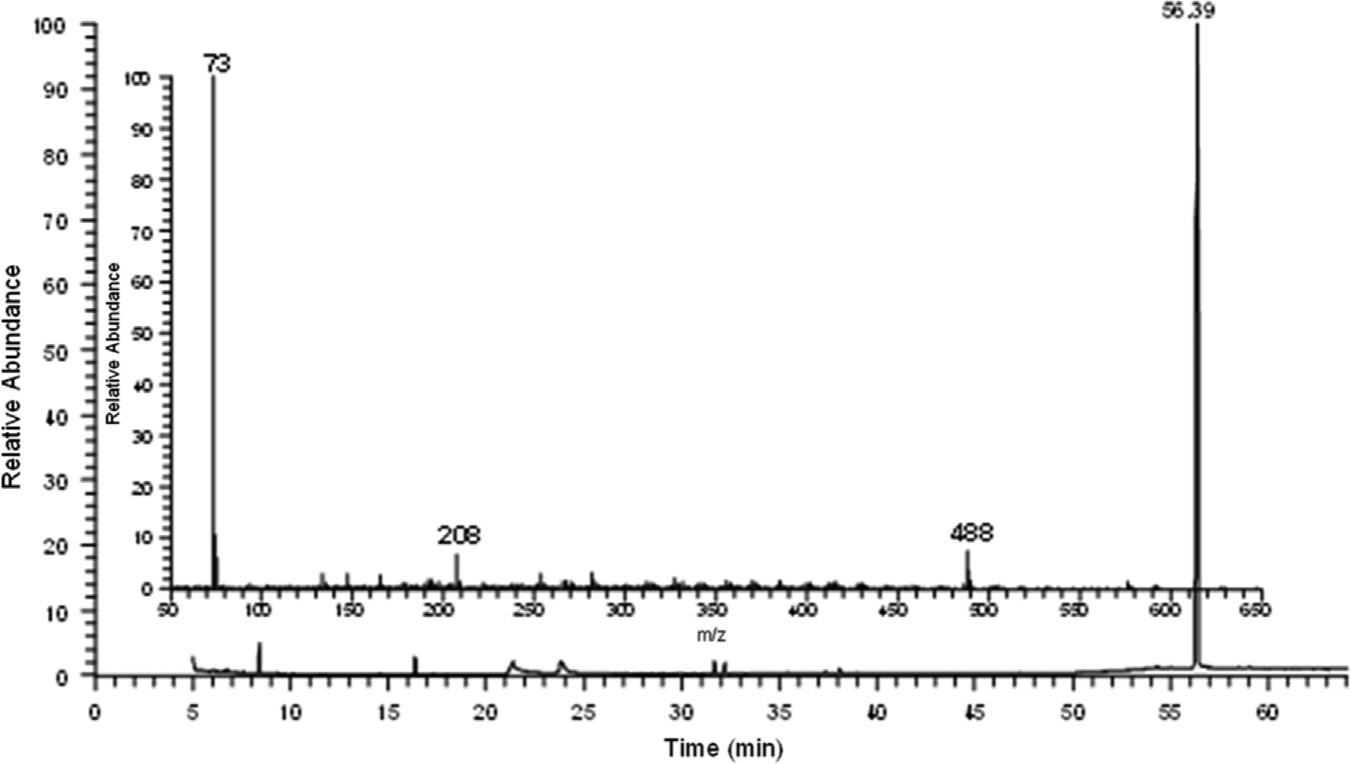
GC–MS chromatogram of products of ellagic acid methanolysis after silylation with the respective mass spectrum of the peak at the retention time 56.39 min.

The data presented in Section 1.1 include gas chromatography-mass spectrometry (GC–MS) chromatograms and respective GC–MS spectra of silylated methanolysis products obtained from phenolic compounds standards such as 4-hydroxycinnamic, ferulic and veratric acids (Fig. 1), 5-*O*-caffeoylquinic acid (Fig. 2), hesperidin (Fig. 3), naringin (Fig. 4), and ellagic acid (Fig. 5).

In Section 1.2 are presented data on the chemical composition of Arabica and Robusta coffee beans, including chlorogenic acid composition (Table 1), simple sugars, caffeine, and protein contents (Table 2) and total sugar composition (Table 3), but also the chromatic properties of respective coffee powders (Table 4). Moreover, data on the chemical composition of high molecular weight materials (HMWMs) isolated from roasted Arabica and Robusta coffee infusions are presented in Subsection 1.2 in Table 5, Table 6. The latter includes the phenolic compounds and quinic acid released by methanolysis from coffee HMWMs.

**Table 1:** Chlorogenic acid (CGA) composition (g/100 g of green or roasted coffee).

CGA	Green coffees		Roasted coffees	
	Arabica	Robusta	Arabica	Robusta
3-*O*-caffeoylquinic acid (3-CQA)	0.501±0.034	0.666±0.025	0.079±0.007	0.090±0.000
1-*O*-feruloylquinic acid (1-FQA)	0.059±0.000	0.059±0.000	0.018±0.000	0.043±0.000
3-*O*-coumaroylquinic acid (3-CoQA)	–	–	0.073±0.000	0.080±0.000
5-*O*-caffeoylquinic acid (5-CQA)	4.353±0.253	5.061±0.295	1.492±0.143	1.517±0.095
3-*O*-feruloylquinic acid (3-FQA)	0.061±0.007	0.072±0.006	0.654±0.002	0.661±0.038
4-*O*-caffeoylquinic acid (4-CQA)	0.657±0.001	0.932±0.031	0.029±0.005	0.014±0.001
5-*O*-coumaroylquinic acid (5-CoQA)	0.098±0.004	0.070±0.004	0.182±0.000	0.185±0.000
5-*O*-feruloylquinic acid (5-FQA)	0.369±0.000	0.843±0.000	–	–
4-*O*-feruloylquinic acid (4-FQA)	0.060±0.008	0.059±0.008	–	–
4-*O*-coumaroylquinic acid (4-CoQA)	0.069±0.005	0.070±0.005	–	–
3,4-di-*O*-caffeoylquinic acid (3,4-diCQA)	0.284±0.036	0.760±0.025	0.054±0.000	0.060±0.000
3,5-di-*O*-caffeoylquinic acid (3,5-diCQA)	0.459±0.088	0.798±0.020	0.201±0.029	0.379±0.005
*Total (CGA)*	6.972±0.140	9.397±0.346	2.781±0.209	3.029±0.192

**Table 2:** Simple sugars, caffeine, and protein contents (g/100 g of green or roasted coffee).

Coffee samples	Simple sugars				Caffeine	Protein^a^
	Sucrose	Glucose	Fructose	Total		
*Green coffees*						
Arabica	7.14±0.53	0.02±0.00	0.03±0.01	7.19±0.53	1.05±0.05	11.68±0.92
Robusta	4.49±0.00	0.07±0.01	0.12±0.01	4.67±0.02	2.57±0.02	12.97±0.83
*Roasted coffees*						
Arabica	0.14±0.00	0.05±0.00	0.01±0.00	0.20±0.00	0.88±0.05	13.34±0.98
Robusta	0.04±0.00	0.02±0.00	0.01±0.00	0.07±0.00	1.59±0.17	16.08±1.18

a (N_total_ – N_caffeine_) × 6.25

**Table 3:** Total sugar composition (g/100 g of green or roasted coffee).

Coffee samples	Sugars						Total	Total_Polymeric_^b^
	Rhamnose	Arabinose	Galactose	Mannose	Glucose	Glc_Polymeric_^a^		
*Green coffees*								
Arabica	0.04±0.0	2.00±0.24	7.82±0.31	16.2±0.9	9.76±0.20	6.17	35.8±1.6	32.2
Robusta	0.06±0.0	1.98±0.06	9.96±0.30	15.0±1.2	9.00±1.1	6.69	36.0±2.7	33.7
*Roasted coffees*								
Arabica	0.02±0.0	1.15±0.16	6.25±1.07	16.9±2.5	6.11±1.01	5.99	30.5±4.7	30.4
Robusta	0.06±0.0	1.53±0.11	8.96±0.26	15.5±0.1	6.12±0.36	6.08	32.2±0.3	32.2

a Glc_Polymeric_ – polymeric glucose, determined by subtracting to the total glucose content the contribution of glucose present as free glucose and sucrose;

b Total_Polymeric_ – polymeric sugars, determined by subtracting to the total sugar content the contribution of glucose present as free glucose and sucrose.

**Table 4:** Chromatic properties of roasted coffee powders.^a^

Coffee samples	L*	a*	b*	C*	h*
Arabica	37.628±0.742	9.966±0.113a	15.746±0.328	18.635±0.330	56.669
Robusta	40.406±0.210	9.864±0.112a	18.984±0.571	21.394±0.554	62.544

a Identical letters in the same column indicate no statistically significant differences.

**Table 5:** Yield, chemical composition and spectroscopic properties of the high molecular weight material (HMWM) isolated from roasted Arabica and Robusta coffee infusions.^a^

	Arabica	Robusta
Yield (g /100 g coffee)	5.69	7.63
Rhamnose (Rha)	0.21±0.02	0.77±0.05
Arabinose (Ara)	3.47±0.07	4.67±0.25
Galactose (Gal)	28.2±1.3	32.7±3.8
Mannose (Man)	4.51±0.46	7.62±0.94
Glucose (Glc)	0.82±0.10	0.53±0.03
Total sugars	37.2±0.6a	46.2±5.1b
Protein	11.3±0.4a	12.3±1.0a
*K*_mix 280 nm_	6.49±0.52a	7.49±0.46a
*K*_mix 325 nm_	5.34±0.38a	6.30±0.37b
*K*_mix 405 nm_	2.72±0.22^a^	2.87±0.18a
Melanoidin brown index (MBI)	5.28	6.93

a For each chemical component, identical letters in the same row indicate no statistically significant differences (t-Student test, *p*<0.05); *K*_mix_ – specific extinction coefficient.

**Table 6:** Phenolic compounds and quinic acid (mmol/100 g) released from HMWM isolated from roasted Arabica and Robusta coffee infusions.^a^

	Arabica	Robusta
***Adsorbed***		
3-*O*-caffeoylquinic acid (3-CQA)	0.011±0.002	0.010±0.004
3-*O*-coumaroylquinic acid (3-CoQA)	0.005±0.001	0.011±0.006
5-*O*-caffeoylquinic acid (5-CQA)	0.015±0.002	0.017±0.004
3-*O*-feruloylquinic acid (3-FQA)	0.022±0.001	0.050±0.002
5-*O*-coumaroylquinic acid (5-CoQA)	0.012±0.001	0.006±0.001
3,4-di-*O*-caffeoylquinic acid (3,4-diCQA)	0.008±0.001	0.006±0.001
3,5-di-*O*-caffeoylquinic acid (3,5-diCQA)	0.005±0.001	0.007±0.001
*Total (adsorbed phenolics)*	0.078±0.002§.a	0.107±0.011§.a
***Saponification***		
Caffeic acid	1.74±0.02	2.89±0.078
4-Hydroxycinnamic acid	0.030±0.001	0.029±0.000
Ferulic acid	0.161±0.000	0.407±0.029
*Total (phenolics released by saponification)*	1.93±0.11§.a	3.33±0.02§.a
***Methanolysis***		
Caffeic acid	11.6±2.5	17.1±1.6
4-Hydroxycinnamic acid	n.d	n.d
Ferulic acid	2.0±0.3	2.1±0.3
*Total (phenolics released by methanolysis)*	13.6±4.1§	19.2±1.3§
Quinic acid	4.8±0.3	11.8±1.5
***Alkaline fusion***		
Gallic acid	0.200±0.006	0.305±0.056
Hydroquinone	1.80±0.27	3.23±0.37
3,4-Dihydroxybenzoic acid	10.6±4.2	19.0±0.3
4-Hydroxybenzoic acid	9.30±4.85	17.0±3.6
3,5-Dihydroxybenzoic acid	5.46±0.88	3.86±0.20
2,3-Dihydroxybenzoic acid	0.928±0.055	1.21±0.05
Benzoic acid	2.18±0.09	0.965±0.389
Salicylic acid	1.77±0.17	3.68±2.84
*Total (phenolics released by alkaline fusion)*	32.3±2.3	49.3±3.5
**ANOVA**		%Variation
Interaction	*p*<0.0001 (4)	3.9
Coffee variety	*p*<0.0001 (1)	3.2
Phenolic method	*p*<0.0001 (4)	91.8

a Rows with the same symbol (§) and columns with the same letter indicate no statistically significant differences (*p*<0.05). Tuckey post-hoc test.

**Fig. 6:**
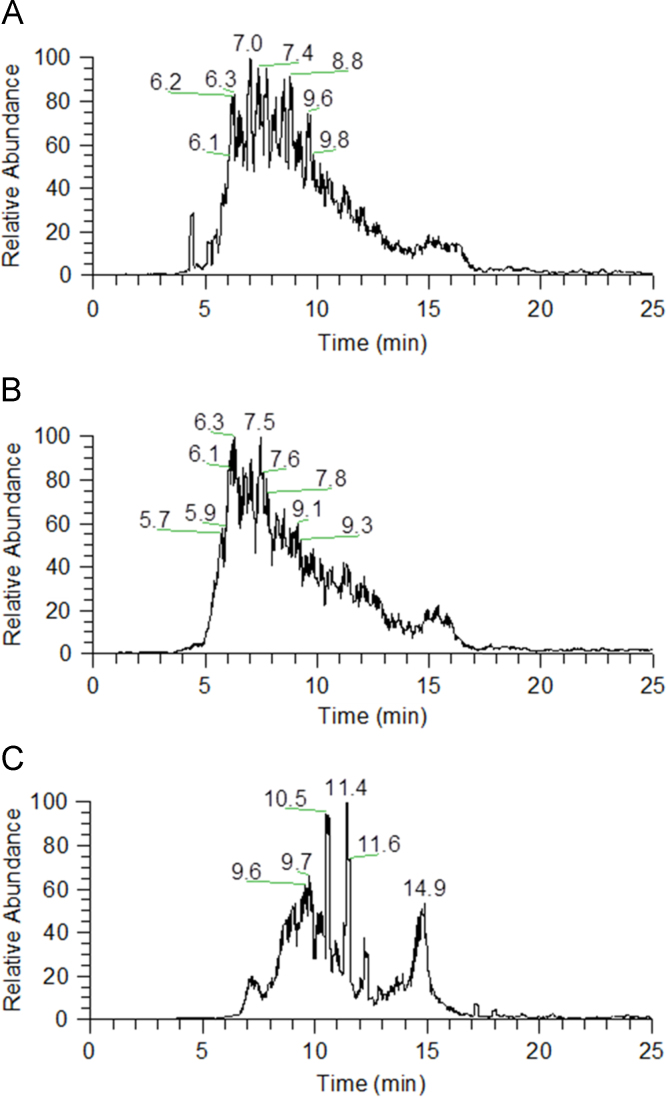
LC-MS reconstructed ion chromatograms (RICs) of the [M+H]^+^ ions of A) HexYL (*m/z* 457) and B) Hex_2_YL (*m/z* 619), acquired from the roasted Man_3_-CQA-YL mixture, and C) HexLY (*m/z* 457), acquired from the roasted Man_3_-LY mixture.

The data presented in Section 1.3 include the mass losses observed after roasting of model mixtures prepared using commercial standards of coffee related carbohydrates, phenolic compounds and peptides (Table 7). The commercial standards used as models of coffee bean components were as follow: (β1→4)-d-mannotriose (Man_3_), an oligosaccharide structurally related to the backbone of coffee galactomannans; 5-*O*-caffeoylquinic acid (5-CQA), the most abundant phenolic compound in green coffee beans; and dipeptides composed by tyrosine (Y) and leucine (L), used as models of coffee proteins. Additionally, malic acid (MalA) and citric acid (CitA), the most abundant aliphatic acids present in green coffee beans, were also used. In Subsection 1.3 are also presented electrospray mass spectrometry (ESI-MS) data obtained from the model mixtures, either by direct infusion of the sample into the mass spectrometer, or online coupling to liquid chromatography (LC). Fig. 6 shows LC-MS reconstructed ion chromatograms (RICs) acquired from the roasted mixture Man_3_-CQA-YL. Table 8 contains the detailed identification of all the ions observed by LC-MS analysis of roasted Man_3_ and mixtures Man_3_-CQA-YL, Man_3_-CQA, Man_3_-YL, and Man_3_-LY. In Table 9 are presented the accurate masses obtained from high resolution and high mass accuracy measurements using a LTQ-Orbitrap mass spectrometer for the ions identified after roasting of the mixture Man_3_-CQA. Table 10, Table 11 summarize the ions identified by ESI-MS analysis of the roasted mixtures Man_3_-MalA and Man_3_-CitA, respectively. In Table 12 the accurate masses found by LTQ-Orbitrap for the ions identified after roasting of the mixture Man_3_-YL are presented. Table 13 provides data on the LC-MS^2^ fragmentation of roasting-induced compounds formed from the mixture Man_3_-YL.

**Table 7:** Total mass loss (%) and mass loss between 150–175 °C (%) during thermal processing of Man_3_ and mixtures Man_3_-CQA-YL, Man_3_-CQA, Man_3_-MalA, Man_3_-CitA, Man_3_-YL, and Man_3_-LY, and, and color of the corresponding resulting material.

Sample	Total mass loss	Mass loss between 150–175 °C	Color of the resulting material
Man_3_	7.1	–	White
Man_3_-CQA-YL	12.7	4.3	Dark brown
Man_3_-CQA	10.7	2.2	Brown
Man_3_-MalA	7.6	3.1	Light brown
Man_3_-CitA	12.4	3.6	Light brown
Man_3_-YL	14.9	5.2	Dark brown
Man_3_-LY	14.5	6.5	Dark brown

**Table 8:** Summary of [M+Na]^+^ and [M+H]^+^ ions identified by LC-MS analysis after roasting (T1) of the Man_3_ and mixtures Man_3_-CQA-YL, Man_3_-CQA, Man_3_-YL, and Man_3_-LY, with the indication of the *m/z* values and the proposed assignments.

Proposed assignment	Number of hexose (Hex) units (*n*)								
	1	2	3	4	5	6	7	8	9
**Roasted Man**_**3**_									
[Hex_*n*_+Na]^+^		365	527	689					
**Roasted Man**_**3**_**-CQA-YL**^a^									
[Hex_*n*_+Na]^+^		365	527	689					
[Hex_*n*_-H_2_O+Na]^+^		347	509						
[Hex_*n*_-2H_2_O+Na]^+^		329	491						
[Hex_*n*_-3H_2_O+Na]^+^		311	473						
[YL_*n*_+H]^+^	295								
[YL_*n*_-H_2_O+H]^+^	277								
[YL_*n*_-NH_3_+O+H]^+^	294								
[(CQA)_*n*_+Na]^+^	377								
[YL_*n*_(CQA)+H]^+^	631								
[Hex_*n*_YL+H]^+^	457	619	781	943	1105				
[Hex_*n*_YL-H_2_O+H]^+^	439	601	763	925	1087				
[Hex_*n*_YL-2H_2_O+H]^+^	421	583	745	907	1069				
[Hex_*n*_YL-3H_2_O+H]^+^	403	565	727	889					
[Hex_*n*_YL-4H_2_O+H]^+^	385	547	709	871					
[Hex_*n*_(YL)_2_+H]^+^	733	895	1057						
[Hex_*n*_CQAYL+H]^+^	793	955	1117						
**Roasted Man**_**3**_**-CQA**^a^									
[Hex_*n*_+Na]^+^		365	527	689	851	1013	1175	1337	1499
[Hex_*n*_-H_2_O+Na]^+^		†347	†509	671	833	995	1157	1319	
[Hex_*n*_-2H_2_O+Na]^+^		329	491	653					
[Hex_n_-3H_2_O+Na]^+^		311	473	635					
[(CQA)_*n*_+Na]^+^	377†	713							
[(CQA)_*n*_CA+Na]^+^	539‡								
[(CQA)_*n*_CA-H_2_O+Na]^+^	521‡								
[Hex_*n*_CQA+Na]^+^	539‡	701†	863	1025	1187	1349			
[Hex_*n*_CQA-H_2_O+Na]^+^	521‡	683†							
[Hex_*n*_CQA-3H_2_O+Na]^+^	485	647							
[Hex_*n*_(CQA)_2_+Na]^+^	875								
[Hex_*n*_QA+Na]^+^	377†	539‡	701†						
[Hex_*n*_QA-H_2_O+Na]^+^		521‡	683†						
[Hex_*n*_CA-H_2_O+Na]^+^	†347	†509							
**Roasted Man**_**3**_**-YL**^a^									
[Hex_*n*_+Na]^+^	203	365	527	689	851	1013			
[Hex_*n*_-H_2_O+Na]^+^		347	509						
[Hex_*n*_-2H_2_O+Na]^+^		329	491						
[Hex_*n*_-3H_2_O+Na]^+^		311	473						
[(YL)_*n*_+H]^+^	295								
[(YL)_*n*_-H_2_O+H]^+^	277								
[(YL)_*n*_-NH_3_+O+H]^+^	294								
[Hex_*n*_YL+H]^+^	457	619	781	943	1105	1267			
[Hex_*n*_YL-H_2_O+H]^+^	439	601	763	925	1087	1249			
[Hex_*n*_YL-2H_2_O+H]^+^	421	583	745	907					
[Hex_*n*_YL-3H_2_O+H]^+^	403	565	727	889					
[Hex_*n*_YL-4H_2_O+H]^+^	385	547	709	871					
[Hex_*n*_(YL)_2_+H]^+^	733	895	1057						
**Roasted Man**_**3**_**-LY**									
[Hex_*n*_+Na]^+^	203	365	527	689	851				
[Hex_*n*_-H_2_O+Na]^+^		347	509	671	833				
[Hex_*n*_-2H_2_O+Na]^+^		329	491						
[Hex_*n*_-3H_2_O+Na]^+^		311	473						
[(LY)_*n*_+H]^+^	295								
[(LY)_*n*_-H_2_O+H]^+^	277								
[(LY)_*n*_-NH_3_+O+H]^+^	294								
[Hex_*n*_LY+H]^+^	457	619	781	943	1105	1267			
[Hex_*n*_LY-H_2_O+H]^+^	439	601	763	925	1087	1249			
[Hex_*n*_LY-2H_2_O+H]^+^	421	583	745	907	1069				
[Hex_*n*_LY-3H_2_O+H]^+^	403	565	727	889					
[Hex_*n*_LY-4H_2_O+H]^+^	385	547	709	871					
[Hex_*n*_(LY)_2_+H]^+^	733	895	1057						

a Ion assignment supported by accurate masses found by LTQ-Orbitrap for roasted mixtures Man_3_-CQA and Man_3_-YL. The ions marked with the symbol † or ‡ were attributed to different isobaric compounds: † for two and ‡ for three possible compounds. For roasted Man_3_-CQA-YL, the ion assignment was made considering the most abundant isobaric compounds identified in the roasted mixture Man_3_-CQA. However, the presence of isobars in roasted Man_3_-CQA-YL cannot be excluded.

**Table 9:** Accurate masses found by LTQ-Orbitrap for the ions identified after roasting of the mixture Man_3_-CQA. The theoretical mass and the difference between the theoretical and experimental masses for each predicted formula were obtained from Xcalibur software.

Experimental mass (*m/z*)	Theoretical mass (*m/z*)	Mass error (ppm)	RDB equiv.	Composition	Proposed assignment
323.0757	323.0761	−1.28	8.5	C_15_H_15_O_8_	[HexCA-H_2_O-H]^−^
323.097	323.0973	−0.94	3.5	C_12_H_19_O_10_	[Hex_2_-H_2_O-H]^−^
341.1074	341.1078	−1.17	2.5	C_12_H_21_O_11_	[Hex_2_-H]^-^
353.0862	353.0867	−1.41	8.5	C_16_H_17_O_9_	[CQA-H]^−^
353.1072	353.1078	−1.75	3.5	C_13_H_21_O_11_	[HexQA-H]^−^
461.1071	461.1078	−1.51	12.5	C_22_H_21_O_11_	[HexCQA-3H_2_O-H]^−^
497.1071	497.1078	−1.46	15.5	C_25_H_21_O_11_	[(CQA)CA-H_2_O-H]^−^
497.1281	497.129	−1.76	10.5	C_22_H_25_O_13_	[HexCQA-H_2_O-H]^−^
497.1492	497.1501	−1.86	5.5	C_19_H_29_O_15_	[Hex_2_QA-H_2_O-H]−
503.1599	503.1607	−1.61	3.5	C_18_H_31_O_16_	[Hex_3_-H]^−^
515.1182	515.1184	−0.37	14.5	C_25_H_23_O_12_	[(CQA)CA-H]^−^
515.1385	515.1395	−1.96	9.5	C_22_H_27_O_14_	[HexCQA-H]^−^
515.1596	515.1607	−2.02	4.5	C_19_H_31_O_16_	[Hex_2_QA-H]^−^
623.1594	623.1607	−2.02	13.5	C_28_H_31_O_16_	[Hex_2_CQA-3H_2_O-H]^−^
659.1805	659.1818	−1.97	11.5	C_28_H_35_O_18_	[Hex_2_CQA-H_2_O-H]^−^
659.2029	659.2029	−0.09	6.5	C_25_H_39_O_20_	[Hex_3_QA-H_2_O-H]−
677.1908	677.1924	−2.3	10.5	C_28_H_37_O_19_	[Hex_2_CQA-H]^−^
677.2121	677.2135	−1.97	5.5	C_25_H_41_O_21_	[Hex_3_QA-H]−
689.1703	689.1712	−1.4	16.5	C_32_H_33_O_17_	[(CQA)_2_-H]^−^
839.2433	839.2452	−2.29	11.5	C_34_H_47_O_24_	[Hex_3_CQA-H]^−^
851.222	851.224	−2.41	17.5	C_38_H_43_O_22_	[Hex(CQA)_2_-H]^−^
1001.2961	1001.298	−1.9	12.5	C_40_H_57_O_29_	[Hex_4_CQA-H]−
1163.3479	1163.3508	−2.51	13.5	C_46_H_67_O_34_	[Hex_5_CQA-H]^−^
1325.3998	1325.4036	−2.92	14.5	C_52_H_77_O_39_	[Hex_6_CQA-H]^−^

**Table 10:** Summary of [M+Na]^+^ ions identified by ESI-MS analysis after roasting of the mixture Man_3_-MalA, with the indication of the *m/z* values and the proposed assignments.

Proposed assignment	Number of hexose (Hex) units (*n*)								
	1	2	3	4	5	6	7	8	9
[Hex_*n*_+Na]^+^	203	365	527	689	851	1013	1175	1337	1499
[Hex_*n*_-H_2_O+Na]^+^	185	347	509	671	833	995	1157	1319	1481
[(MalA)_*n*_+Na]^+^	157								
[Hex_*n*_MalA+Na]^+^	319	481	643	805	967	1129	1291	1453	
[Hex_*n*_MalA-H_2_O+Na]^+^	301	463	625	787	949	1111	1273	1435	
[Hex_*n*_(MalA)_2_+Na]^+^	435	597	759	921	1083	1245	1407

**Table 11:** Summary of [M+Na]^+^ ions identified by ESI-MS analysis after roasting of the mixture Man_3_-CitA, with the indication of the *m/z* values and the proposed assignments.

Proposed assignment	Number of hexose (Hex) units (*n*)								
	1	2	3	4	5	6	7	8	9
[Hex_*n*_+Na]^+^	203	365	527	689	851	1013	1175	1337	1499
[Hex_*n*_-H_2_O+Na]^+^	185	347	509	671	833	995	1157	1319	1481
[(CitA)_*n*_+Na]^+^	215								
[Hex_*n*_CitA+Na]^+^	377	539	701	863	1025	1187	1349		
[Hex_*n*_CitA-H_2_O+Na]^+^	359	521	683	845	1007	1169	1331		
[Hex_*n*_(CitA)_2_+Na]^+^	551	713	875	1037	1199	1361

**Table 12:** Accurate masses found by LTQ-Orbitrap for the ions identified after roasting of the mixture Man_3_-YL. The theoretical mass and the difference between the theoretical and experimental masses for each predicted formula were obtained from Xcalibur software.

Experimental mass (*m/z*)	Theoretical mass (*m/z*)	Mass error (ppm)	RDB equiv.	Composition	Proposed assignment
275.1393	275.139	0.91	7.5	C_15_H_19_O_3_N_2_	[YL-H_2_O-H]^−^
292.1184	292.1179	1.58	7.5	C_15_H_18_O_5_N	[YL-NH_3_+O-H]^−^
293.15	293.1496	1.32	6.5	C_15_H_21_O_4_N_2_	[YL-H]^−^
341.1082	341.1078	1.18	2.5	C_12_H_21_O_11_	[Hex_2_-H]^−^
383.1606	383.1601	1.15	11.5	C_21_H_23_O_5_N_2_	[HexYL-4H_2_O-H]^−^
401.171	401.1707	0.69	10.5	C_21_H_25_O_6_N_2_	[HexYL-3H_2_O-H]^−^
419.1815	419.1813	0.6	9.5	C_21_H_27_O_7_N_2_	[HexYL-2H_2_O-H]^−^
437.1921	437.1918	0.59	8.5	C_21_H_29_O_8_N_2_	[HexYL-H_2_O-H]^−^
455.2027	455.2024	0.67	7.5	C_21_H_31_O_9_N_2_	[HexYL-H]^−^
503.1609	503.1607	0.55	3.5	C_18_H_31_O_16_	[Hex_3_-H]^−^
545.2133	545.213	0.53	12.5	C_27_H_33_O_10_N_2_	[Hex_2_YL-4H_2_O-H]^−^
563.2237	563.2235	0.34	11.5	C_27_H_35_O_11_N_2_	[Hex_2_YL-3H_2_O-H]^−^
581.2342	581.2341	0.12	10.5	C_27_H_37_O_12_N_2_	[Hex_2_YL-2H_2_O-H]^−^
599.2448	599.2447	0.24	9.5	C_27_H_39_O_13_N_2_	[Hex_2_YL-H_2_O-H]^−^
617.255	617.2552	−0.31	8.5	C_27_H_41_O_14_N_2_	[Hex_2_YL-H]^−^
707.266	707.2658	0.25	13.5	C_33_H_43_O_15_N_2_	[Hex_3_YL-4H_2_O-H]^−^
725.2764	725.2764	0.11	12.5	C_33_H_45_O_16_N_2_	[Hex_3_YL-3H_2_O-H]^−^
731.3499	731.3498	0.16	13.5	C_36_H_51_O_12_N_4_	[Hex(YL)_2_-H]^−^
743.287	743.2869	0.13	11.5	C_33_H_47_O_17_N_2_	[Hex_3_YL-2H_2_O-H]^−^
761.2977	761.2975	0.25	10.5	C_33_H_49_O_18_N_2_	[Hex_3_YL-H_2_O-H]^−^
779.3081	779.3081	0.03	9.5	C_33_H_51_O_19_N_2_	[Hex_3_YL-H]^−^
869.3182	869.3186	−0.49	14.5	C_39_H_53_O_20_N_2_	[Hex_4_YL-4H_2_O-H]^−^
887.3288	887.3292	−0.41	13.5	C_39_H_55_O_21_N_2_	[Hex_4_YL-3H_2_O-H]^−^
905.3395	905.3397	−0.31	12.5	C_39_H_57_O_22_N_2_	[Hex_4_YL-2H_2_O-H]^−^
923.3493	923.3503	−1.05	11.5	C_39_H_59_O_23_N_2_	[Hex_4_YL-H_2_O-H]^−^
941.3579	941.3609	−3.15	10.5	C_39_H_61_O_24_N2	[Hex_4_YL-H]^−^
1085.4015	1085.4031	−1.49	12.5	C_45_H_69_O_28_N_2_	[Hex_5_YL-H_2_O-H]^−^
1247.454	1247.456	−1.54	13.5	C_51_H_79_O_33_N_2_	[Hex_6_YL-H_2_O-H]^−^

**Table 13:** Compounds identified after roasting of the mixture Man_3_-YL: the *m/z* values of the ions identified, the proposed assignments, the retention time (RT), and the most abundant product ions observed in the respective LC-MS^2^ spectrum, with the indication of the *m/z* values, mass differences relative to the precursor ion, and the identification of the most informative product ions.

*m/z*	Assignment	RT	LC-MS^2^
203	[Hex+Na]^+^	4.0	aNo LC-MS^2^ spectrum
277	[YL-H_2_O+H]^+^	10.4–15.6	249 (-28), 136 (-141, a_1_), 171 (-106)
294	[YL-NH_3_+O+H]^+^	17.7–29.9	248 (-46), 276 (-18), 132 (-162, -(Y-NH_3_+O)_res_, [L+H]^+^), 266 (-28), 220 (-74)
295	[YL+H]^+^	7.2–16.6	136 (-159, a_1_), 278 (-17, -NH_3_), 249 (-46, -HCO_2_H), 119 (-176, a_1_-NH_3_)
311	[Hex_2_-3H_2_O+Na]^+^	4.3	185 (-126, -(Hex-3H_2_O)), 149 (-162, -Hex_res_)
329	[Hex_2_-2H_2_O+Na]^+^	3.9	167 (-162, -Hex_res_), 185 (-144, -(Hex-2H_2_O)), 203 (-126, -(Hex-2H_2_O)_res_)
347	[Hex_2_-H_2_O+Na]^+^	4.1	329 (-18), 287 (-60), 185 (-162, -Hex_res_), 203 (-144, -(Hex-H_2_O)_res_)
365	[Hex_2_+Na]^+^	4.2	347 (-18), 305 (-60), 203 (-162, -Hex_res_), 185 (-180, -Hex)
385	[HexYL-4H_2_O+H]^+^	35.6	339 (-46), 367 (-18), 357 (-28), 226 (-159, (a_1_+(Hex-4H_2_O)_res_)), 311 (-74),
			254 (-131, -L)
385	[HexYL-4H_2_O+H]^+^	41.1	339 (-46), 367 (-18), 357 (-28), 226 (-159, (a_1_+(Hex-4H_2_O)_res_)), 311 (-74),
			254 (-131, -L)
403	[HexYL-3H_2_O+H]^+^	17.6	385 (-18), 244 (-159, (a_1_+(Hex-3H_2_O)_res_)), 126 (-277), 357 (-46), 278 (-125)
403	[HexYL-3H_2_O+H]^+^	25.1	272 (-131, -L), 244 (-159, (a_1_+(Hex-3H_2_O)_res_)), 385 (-18), 357 (-46), 279 (-124)
421	[HexYL-2H_2_O+H]^+^	18.1	290 (-131, -L), 262 (-159, (a_1_+(Hex-2H_2_O)_res_)), 403 (-18), 393 (-28), 375 (-46)
421	[HexYL-2H_2_O+H]^+^	20.2	290 (-131, -L), 262 (-159, (a_1_+(Hex-2H_2_O)_res_)), 403 (-18), 375 (-46), 391 (-30)
421	[HexYL-2H_2_O+H]^+^	29.9	262 (-159, (a_1_+(Hex-2H_2_O)_res_)), 403 (-18), 290 (-131, -L), 244 (-177)
439	[HexYL-H_2_O+H]^+^	18.0	280 (-159, (a_1_+(Hex-H_2_O)_res_)), 393 (-46), 295 (-144, -(Hex-H_2_O)_res_, [YL+H]^+^)
457	[HexYL+H]^+^	5.7–16.9	439 (-18), 373 (-84), 421 (-36), 307 (-150), 295 (-162, -Hex_res_, [YL+H]^+^), 403 (-54), 136 (-321, a_1_), 298 (-159, (a_1_+Hex_res_))
473	[Hex_3_-3H_2_O+Na]^+^	4.2	347 (-126, -(Hex-3H_2_O)), 311 (-162, -Hex_res_)
491	[Hex_3_-2H_2_O+Na]^+^	4.1	329 (-162, -Hex_res_), 347 (-144, -(Hex-2H_2_O)), 365 (-126, -(Hex-2H_2_O)_res_)
509	[Hex_3_-H_2_O+Na]^+^	4.2	347 (-162, -Hex_res_), 491 (-18), 449 (-60), 365 (-144, -(Hex-H_2_O)_res_), 185 (-324, -2xHex_res_)
527	[Hex_3_+Na]^+^	4.2	365 (-162, -Hex_res_), 347 (-180, -Hex), 509 (-18), 467 (-60), 185 (-342, [Hex_res_+Na]^+^)
547	[Hex_2_YL-4H_2_O+H]^+^	18.4–24.9	529 (-18), 388 (-159, (a_1_+(Hex_2_-4H_2_O)_res_)), 501 (-46), 385 (-162, -(Hex-H_2_O)),
			416 (-131, -L), 511 (-36), 421 (-126, -(Hex-3H_2_O)), 403 (-144, -(Hex-3H_2_O)_res_)
565	[Hex_2_YL-3H_2_O+H]^+^	16.5	288 (-277), 547 (-18), 403 (-162, -Hex_res_), 406 (-159, a_1_+(Hex_2_-3H_2_O)_res_), 529 (-36)
565	[Hex_2_YL-3H_2_O+H]^+^	19.6–21.2	547 (-18), 406 (-159, a_1_+(Hex_2_-3H_2_O)_res_), 403 (-162, -Hex_res_), 529 (-36),
			439 (-126, -(Hex-3H_2_O))
583	[Hex_2_YL-2H_2_O+H]^+^	24.1	421 (-162, -Hex_res_), 565 (-18), 262 (-321, a_1_+(Hex-2H_2_O)_res_)), 290 (-293, -(Hex_res_+L), 403 (-180, -Hex)
601	[Hex_2_YL-H_2_O+H]^+^	16.9	439 (-162, -Hex_res_), 280 (-321, a_1_+(Hex-H_2_O)_res_)), 295 (-306, [YL+H]^+^),
			393 (-208, -(Hex_res_+HCO_2_H))
619	[Hex_2_YL+H]^+^	5.8–16.4	457 (-162, -Hex_res_), 601 (-18), 307 (-312, -(Hex_res_+150)), 373 (-246, -(Hex_res_+84))
689	[Hex_4_+Na]^+^	3.9	527 (-162, -Hex_res_), 365 (-324, -2xHex_res_), 203 (-486, -3xHex_res_)
709	[Hex_3_YL-4H_2_O+H]^+^	15.7–21.1	547 (-162, -Hex_res_), 691 (-18), 635 (-74), 529 (-180, -Hex), 583 (-126, -(Hex-3H_2_O)), 550 (-159, (a_1_+(Hex_3_-4H_2_O)_res_))
727	[Hex_3_YL-3H_2_O+H]^+^	16.6–20.6	565 (-162, -Hex_res_), 709 (-18), 403 (-324, -2xHex_res_), 547 (-180, -Hex), 295 (-270, [YL+H]^+^)
733	[Hex(YL)_2_+H]^+^	26.3	439 (-294, -YL), 280 (-453), 602 (-131, -L), 574 (-159), 393 (-340), 715 (-18),
			295 (-438, [YL+H]^+^), 571 (-162, -Hex_res_)
733	[Hex(YL)_2_+H]^+^	31.6	602 (-131, -L), 439 (-294, -YL), 280 (-453), 393 (-340), 715 (-18), 574 (-159),
			295 (-438, [YL+H]^+^), 571 (-162, -Hex_res_)
745	[Hex_3_YL-2H_2_O+H]^+^	22.3	421 (-324, -2xHex_res_), 583 (-162, -Hex_res_), 262 (-483, a_1_+(Hex-2H_2_O)_res_)),
			403 (-342, -(Hex_res_+Hex)), 290 (-455, -((2xHex_res_)+L))
745	[Hex_3_YL-2H_2_O+H]^+^	23.2	421 (-324, -2xHex_res_), 583 (-162, -Hex_res_), 403 (-342, -(Hex_res_+Hex)),
			262 (-483, a_1_+(Hex-2H_2_O)_res_)), 290 (-455, -((2xHex_res_)+L))
763	[Hex_3_YL-H_2_O+H]^+^	16.7	601 (-162, -Hex_res_), 439 (-324, -2xHex_res_), 280 (-483, (a_1_+(Hex-H_2_O)_res_)), 745 (-18), 295 (-468, [YL+H]^+^)
763	[Hex_3_YL-H_2_O+H]^+^	18.7	439 (-324, -2xHex_res_), 601 (-162, -Hex_res_), 280 (-483, (a_1_+(Hex-H_2_O)_res_)), 745 (-18), 393 (-370, -((2xHex_res_)+HCO_2_H))
781	[Hex_3_YL+H]^+^	5.3–16.2	457 (-324, -2xHex_res_), 619 (-162, -Hex_res_), 373 (-408, -((2xHex_res_)+84),
			298 (-483, (a_1_+Hex_res_))
851	[Hex_5_+Na]^+^	4.2	689 (-162, -Hex_res_), 527 (-324, -2xHex_res_), 671 (-180, -Hex), 365 (-486, -3xHex_res_)
871	[Hex_4_YL-4H_2_O+H]^+^	15.6–21.3	709 (-162, -Hex_res_), 547 (-324, -2xHex_res_), 745 (-126, -(Hex-3H_2_O))
889	[Hex_4_YL-3H_2_O+H]^+^	18.6	727 (-162, -Hex_res_), 565 (-324, -2xHex_res_), 871 (-18), 709 (-180, -Hex), 373 (-516)
889	[Hex_4_YL-3H_2_O+H]^+^	19.8	565 (-324, -2xHex_res_), 727 (-162, -Hex_res_), 871 (-18), 272 (-455, -((2xHex_res_)+L))
889	[Hex_4_YL-3H_2_O+H]^+^	22.1	565 (-324, -2xHex_res_), 403 (-486, -3xHex_res_), 727 (-162, -Hex_res_), 871 (-18),
			709 (-180, -Hex)
895	[Hex_2_(YL)_2_+H]^+^	22.8	733 (-162, -Hex_res_), 439 (-456, -(Hex_res_+YL)), 602 (-293, -(Hex_res_+L)), 764 (-131, -L), 280 (-615), 571 (-324, -2xHex_res_)
895	[Hex_2_(YL)_2_+H]^+^	27.3	733 (-162, -Hex_res_), 602 (-293, -(Hex_res_+L)), 439 (-456, -(Hex_res_+YL)), 764 (-131, -L), 280 (-615), 571 (-324, -2xHex_res_)
907	[Hex_4_YL-2H_2_O+H]^+^	16.8	745 (-162, -Hex_res_), 583 (-324, -2xHex_res_)
907	[Hex_4_YL-2H_2_O+H]^+^	27.4	745 (-162, -Hex_res_), 583 (-324, -2Hex_res_)
925	[Hex_4_YL-H_2_O+H]^+^	16.4	907 (-18), 601 (-324, -2xHex_res_), 763 (-162, -Hex_res_), 889 (-36),
			583 (-342, -(Hex_res_+Hex))
943	[Hex_4_YL+H]^+^	4.3–15.3	619 (-324, -2xHex_res_), 925 (-18), 781 (-162, -Hex_res_), 457 (-486, -3xHex_res_), 373 (-570, -((3xHex_res_)+84)), 295 (-648, -4xHex_res_, [YL+H]^+^)
1013	[Hex_6_+Na]^+^	4.4	851 (-162, -Hex_res_), 527 (-486, -3xHex_res_), 689 (-324, -2xHex_res_), 953 (-60), 833 (-180, Hex)
1057	[Hex_3_(YL)_2_+H]^+^	21.7	733 (-324, -2xHex_res_), 439 (-618, -((2xHex_res_)+YL)), 895 (-162, -Hex_res_),
			602 (-455, -((2xHex_res_)+L))
1057	[Hex_3_(YL)_2_+H]^+^	25.3	733 (-324, -2xHex_res_), 602 (-455, -((2xHex_res_)+L)), 439 (-618, -((2xHex_res_)+YL)), 895 (-162, -Hex_res_), 926 (-131, -L)
1087	[Hex_5_YL-H_2_O+H]^+^	16.2	925 (-162, -Hex_res_), 1069 (-18), 763 (-324, -2xHex_res_)
1105	[Hex_5_YL+H]^+^	4.2–16.7	943 (-162, -Hex_res_), 781 (-324, -2xHex_res_)
1249	[Hex_6_YL-H_2_O+H]^+^	15.9	aNo LC-MS^2^ spectrum
1267	[Hex_6_YL+H]^+^	5.3–16.7	aNo LC-MS^2^ spectrum

a No LC-MS^2^ spectrum, but the ion assignment is corroborated by the observation of other ions of the same series, eluting at a similar retention time (RT). Abbreviations: a_1_ – peptide fragment (Fig. 2A); Hex_res_ – Hexose residue; L – Leucine; L_res_ – Leucine residue; Y – Tyrosine; Y_res_ – Tyrosine residue.

### 1.1 GC–MS data of silylated methanolysis products of phenolic compounds standards

See Fig. 1, Fig. 2, Fig. 3, Fig. 4, Fig. 5–5.

### 1.2 Chemical composition of coffee beans and derived fractions

See Table 1, Table 2, Table 3, Table 4, Table 5, Table 6

### 1.3 Data on the model mixtures mimicking coffee composition

See Fig. 6 and Table 7, Table 8, Table 9, Table 10, Table 11, Table 12, Table 13

Some of the Hex_*n*_ and dehydrated derivatives identified by HPLC-ESI-MS (Table 8) were not observed in the negative ESI-MS spectrum acquired on the LTQ-Orbitrap mass spectrometer (Table 9). This is due to the fact that neutral oligosaccharides ionize better in positive than in negative mode.

The analysis of the reconstructed ion chromatograms (RICs) corroborates the presence of isomeric compounds, i.e. compounds with the same elemental composition but different structures, eluting at different RTs. However, the exact structural differences were not possible to be inferred based on the respective LC-MS^*n*^ spectra (*n*=2–3) because they were very similar, most probably due to the presence of positional isomers. In the case of the compounds bearing a sugar moiety, the structural differences of the isomers can be related to different structures of the sugar moiety, differing on glycosidic linkage positions, and anomeric configuration.

## 2 Experimental design, materials and methods

The methodologies that allowed the data here presented are described in [1] and in cited references. Here, only the protocol for glycosidic linkage analysis is provided, giving a large number of experimental details, usually omitted in research articles due to the words limit.

### 2.1 Glycosidic linkage analysis

A sample (0.5–1 mg) of each unroasted and roasted model (Man_3_ and mixtures) was dissolved with DMSO (1 mL), and then powdered NaOH (40 mg) was added to the solution. After 30 min at room temperature with continuous stirring, samples were methylated by adding of CH_3_I (80 µL), allowed to react 20 min under vigorous stirring. Distilled water (2 mL) was then added, and the solution was neutralized using HCl 1 M. Dichloromethane (3 mL) was then added and, upon vigorous manual shaking and centrifugation, the dichloromethane phase was recovered and washed two times by addition of distilled water (2–3 mL). The organic phase was evaporated to dryness and the resulting material was remethylated using the same procedure. The remethylated material was hydrolyzed with 500 µL of TFA 2 M at 121 °C for 1 h, and the acid was then evaporated to dryness. For carbonyl-reduction, the resulting material was then suspended in 300 µL of NH_3_ 2 M and 20 mg of NaBD_4_ were added. The reaction mixture was incubated at 30 °C for 1 h. After cooling, the excess of borodeuteride was destroyed by the addition of glacial acetic acid (2×50 µL). The partially methylated alditol derivatives were acetylated with acetic anhydride (3 mL) in the presence of 1-methylimidazole (450 μL) during 30 min at 30 °C. To decompose the excess of acetic anhydride, distilled water (3 mL) was added while the tubes were in ice. Dichloromethane (2.5 mL) was then added and, upon vigorous manual shaking and centrifugation, the dichloromethane phase was recovered. The addition of water (3 mL) and dichloromethane (2.5 mL), and the recovery of the organic phase were performed once more. The dichloromethane phase was then washed two times by addition of distilled water (3 mL) and evaporated to dryness. The dried material was dissolved with anhydrous acetone (2 × 1 mL) followed by the evaporation of the acetone to dryness. The partially methylated alditol acetates (PMAAs) were redissolved with anhydrous acetone and identified by gas chromatography-mass spectrometry (GC–MS) on an Agilent Technologies 6890 N Network GC system (Santa Clara, CA) equipped with a DB-1ms column with 30 m of length, 0.25 mm of internal diameter, and 0.1 µm of film thickness (J&W Scientific, Folsom, CA). The GC was connected to an Agilent 5973 Network Mass Selective Detector operating with an electron impact mode at 70 eV, and scanning the *m/z* range 40–500 in a 1 s cycle in a full scan mode acquisition. The oven temperature program used was: initial temperature 50 °C, a linear increase of 8 °C/min up to 140 °C, standing at this temperature for 5 min, followed by linear increase of 0.5 °C/min up to 150 °C, ollowed by linear increase of 40 °C/min up to 250 °C, standing at this temperature for 1 min. The injector and detector temperatures were 220 and 230 °C, respectively. Helium was used as carrier gas at a flow rate of 1.7 mL/min. Relative abundance of each PMAA identified in both unroasted and roasted samples was determined upon integration of each peak using the equipment׳s software.
