# Supplementary material for: Data on coffee composition and mass spectrometry analysis of mixtures of coffee related carbohydrates, phenolic compounds and peptides
Source: Data Brief. 2017 May 17;13:145–61. doi: 10.1016/j.dib.2017.05.027 (PMC5451187; doi:10.1016/j.dib.2017.05.027)
Supplement: Supplementary file 1 — Supplementary material [file mmc1.doc]

**AUTHOR DECLARATION**

Aveiro, 27^th^ April 2017

I confirm that there are no known conflicts of interest associated with this publication.

I confirm that the manuscript has been read and approved by all named authors and that there are no other persons who satisfied the criteria for authorship but are not listed. I further confirm that the order of authors listed in the manuscript has been approved by all of us.

Yours sincerely,

Manuel A. Coimbra
